# The effect of electronic medical records on medication errors, workload, and medical information availability among qualified nurses in Israel– a cross sectional study

**DOI:** 10.1186/s12912-024-01936-7

**Published:** 2024-04-24

**Authors:** Raneen Naamneh, Moran Bodas

**Affiliations:** Department of Emergency & Disaster Management, School of Public Health, Faculty of Medical and Health Sciences, Tel-Aviv University, 39040 Tel-Aviv-Yafo, Israel

**Keywords:** Electronic medical records (EMR), Electronic medication system medication errors, Nurses workload, Medical information availability

## Abstract

**Background:**

Errors in medication administration by qualified nursing staff in hospitals are a significant risk factor for patient safety. In recent decades, electronic medical records (EMR) systems have been implemented in hospitals, and it has been claimed that they contribute to reducing such errors. However, systematic research on the subject in Israel is scarce. This study examines the position of the qualified nursing staff regarding the impact of electronic medical records systems on factors related to patient safety, including errors in medication administration, workload, and availability of medical information.

**Methods:**

This cross-sectional study examines three main variables: Medication errors, workload, and medical information availability, comparing two periods– before and after EMR implementation based on self-reports. A final sample of 591 Israeli nurses was recruited using online private social media groups to complete an online structured questionnaire. The questionnaires included items assessing workload (using the Expanding Nursing Stress Scale), medical information availability (the Carrington-Gephart Unintended Consequences of Electronic Health Record Questionnaire), and medical errors (the Medical Error Checklists). Items were assessed twice, once for the period before the introduction of electronic records and once after. In addition, participants answered open-ended questions that were qualitatively analyzed.

**Results:**

Nurses perceive the EMR as reducing the extent of errors in drug administration (mean difference = -0.92 ± 0.90SD, *p* < 0.001), as well as the workload (mean difference = -0.83 ± 1.03SD, *p* < 0.001) by ∼ 30% on average, each. Concurrently, the systems are perceived to require a longer documentation time at the expense of patients’ treatment time, and they may impair the availability of medical information by about 10% on average.

**Conclusion:**

The results point to nurses’ perceived importance of EMR systems in reducing medication errors and relieving the workload. Despite the overall positive attitudes toward EMR systems, nurses also report that they reduce information availability compared to the previous pen-and-paper approach. A need arises to improve the systems in terms of planning and adaptation to the field and provide appropriate technical and educational support to nurses using them.

**Supplementary Information:**

The online version contains supplementary material available at 10.1186/s12912-024-01936-7.

## Introduction

Clinical/medical error is defined as a preventable adverse effect of medical care, whether it is harmful to the patient or not [1]. One of the most common types of medical error is medication error [2]. These errors seriously threaten individual safety and public health in general and are a challenge for the professionals involved. Such errors are responsible for 7000–9000 deaths per year in the United States of America alone, and the cost of medication errors is estimated at over 40 billion dollars per year, which causes a significant burden on the health system and society [3]. Many people suffer physical and psychological pain due to medication administration errors [2, 3].

In Israel, qualified nurses administer prescription medications to patients staying in hospitals. Many measures are taken to ensure the safety of the process of medication administration in hospitals. According to the medication administration procedure in Israel, published in 2016 by the Ministry of Health [4], every instruction on medication administration should include the date, time, full name of the medication, medication form, dosage, frequency of administration, route of administration, duration of administration, and special instructions if applicable. In addition, administering medication requires the nursing staff to implement a series of actions before administering the treatment itself: address the patient’s sensitivities, compare the details of the instruction with the details of the patient and the medication, pay attention to the patient’s new medication and document the administration of the medication in the patient’s record, specifying the date and time of administration [4].

Unfortunately, despite all the efforts and steps taken by healthcare providers, clinical errors, including medication errors, do happen. Error rates in medication administration are still high, with consequences of significant disability for the victims [2, 5]. Moreover, as a result of these errors, medical staff may experience harm to their self-confidence and work less efficiently, which may lead to more mistakes and further impair patient safety [2, 3].

One way proposed in recent decades to prevent or reduce medication errors is the implementation of Electronic Medical Records (EMR) systems in medical centers [6]. EMR systems include a wide variety of technologies designed to assist medical processes and medical decision-making. EMR is a type of information technology through which doctors and nurses in hospitals can organize large amounts of information about the patient and optimize the use of information in their clinical work [6].

In general, findings in the literature indicate significant advantages of using EMR in improving the quality of patient care. Among the benefits found are improving patient safety, reducing the frequency of errors, saving time, preventing complications, improving communication between caregivers, and improving connectivity to other systems in the hospital, such as the pharmacy, laboratories, imaging centers, and others [7–9]. Many studies have found that computerized medical information systems may reduce errors in drug treatment through correct identification of the patient, increasing the availability of relevant medical information to prevent errors, such as drug interactions, as well as increasing access to current information about the patient’s history of the drug treatment and drug sensitivity [10–24].

However, the findings surrounding the effect of EMR on medical error reduction are still inconclusive with some studies reporting mixed results. For example, in a study conducted at the American University of Beirut Medical Center looking into 2,883 prescriptions, of which 1,475 (51.2%) were from the period before the implementation of electronic prescriptions (paper prescriptions) and 1,408 (48.8%) from the period after the implementation of electronic prescriptions, it was found that electronic prescriptions were associated with a significant reduction in errors in medication dosage and frequency of medication administration. However, they were associated with an increase in duplication errors [19]. Other studies report similar findings [25, 26]. After looking into a decade of data between 2099 and 2018, Classen et al. concluded that (p. 1) *“despite broad adoption and optimization of Electronic Health Record (EHR) systems in hospitals, wide variation in the safety performance of operational EHR systems remains across a large sample of hospitals and EHR vendors, and serious safety vulnerabilities persist in these operational EHRs.”* [27].

Due to the complexity of the management of drug treatment by the nursing staff and the multitude of practices and procedures related to it, studies were carried out examining the subject of the satisfaction of nursing staff with the electronic prescription system. The findings were, again, mixed and inconclusive. Some studies reported an increase in staff satisfaction following gthe introduction of EMRs [28, 29], while others reported dissatisfaction stemming from increased workload and burnout [30, 31] and difficulty in retrieving and accessing information [32]. Consequently, some studies report that EMR systems may slow down and consume valuable time away from treating patients [33].

As evident from the literature, findings are inconclusive, and there is still a need to examine the effectiveness of EMR systems in reducing medication errors, as well as their contribution to patients’ safety and staff functionality. This current study aimed to assess nurses’ perception of EMR systems’ contribution to mitigating medical error, workload, and information availability. The working hypotheses were that nurses perceive the introduction of EMR systems as beneficial to reducing medical errors and workload and increasing information availability, compared with the previous pen & paper system.

## Methods

### STROBE statement

This study adheres to the STROBE (Strengthening the Reporting of Observational Studies in Epidemiology) guidelines.

### Study design

This cross-sectional study was performed between February and May 2022. It focused on a large group of qualified nurses in Israel and compared two periods: before and after implementing EMR systems in hospitals. The participants were asked to answer an online survey evaluating the research variables one time by recollecting the period when analog information collection infrastructures were used (pen-and-paper and printed sheets) and another time by addressing the situation during the current period when computerized medical information systems are being used. Differences between the two periods were analyzed for statistical significance. Of note is that most hospitals in Israel transitioned into electronic medical records by the late 2000s, with the last hospital transitioning in the late 2010s. The introduction of the EMR systems expanded the amount of data collected by the medical staff. For example, tracking of previous hospitalizations and reasons for non-administration of certain drugs are currently being collected in the EMR systems but were not recorded or less recorded in the older pen-and-paper system.

### Population & sampling

The target population for this study was registered nurses in Israel. According to the Israeli Ministry of Health, there are 70,052 registered nurses as of 2020, of which approximately 60,000 have been working before the introduction of EMR Systems [34]. The inclusion criteria for this study were being a registered nurse, an adult (over age 18), Hebrew speaking, and having a recollection of the period in which pen-and-paper records were used. Exclusion criteria were not being a registered nurse, minor, non-Hebrew speaking, and having not worked as a registered nurse during the pen-and-paper period or having no recollection of it.

In the current study, we used non-probability sampling to recruit a relatively large number of participants quickly and affordably. We used social media to recruit the participants, as studies have demonstrated the usefulness of obtaining data through social networks [35]. In addition, the snowball method was used to distribute the questionnaire between colleagues. For the needs of the current research, which requires quick access to a unique (professional) population in a wide geographic distribution, the “snowball” approach was deemed the most practical. In addition, data collection also focused on Hillel-Yafe Medical Center, which experienced a cyber attack in October 2021, causing the entire hospital to resort back to pen-and-paper data management. This created an opportunity to collect data from a more recent occurrence of pen-and-paper utilization in a medical establishment. Emphasis was placed on creating a heterogeneous sample that would represent most of the population by distributing the questionnaire to different groups and cultures, Jews and Arabs.

The minimum sample size was calculated using the WinPepi calculator [36]. According to Aziz et al. [16], the incidence of medication errors reported by EMR system users is 0.5%, compared with 2.5% reported by those using analog (pen and paper) systems. Assuming 95% confidence and 99% power, the minimum sample size required is 100. The final sample in this study included nearly six times more participants (*N* = 591). According to WinPepi’s power calculator for paired samples, given the mean difference reported in this study, the current study has a power of 100%.

### Tools

This study utilized a tool comprising both closed and open-ended questions. The former will be described in the following sub-sections. The latter, representing the qualitative part of this study, was used to understand better nurses’ stances toward EMR systems. This section was constructed of three free text questions: (1) What is your opinion regarding using EMR in your department? (2) In what way does the use of EMR benefit you, if at all? (3) In what way does the use of EMR bother you, if at all? The answers to these questions were analyzed using qualitative methods, according to Shkedi [37]. This approach to qualitative analysis provides a guide to open-text categorization and theme extraction that closely fits with Israeli (Hebrew-speaking audiences.

Participants were first asked to state whether they had the opportunity to experience administering medication while working with pen & paper prescriptions and documents. The lack of such experience was a criterion for exclusion from the study.

**Socio-demographics** were assessed using a questionnaire that included the following variables: gender (nominal; male, female, other), age (continuous; calculated using the year of birth), type of nursing staff (nominal; qualified nurse, department manager, deputy manager), seniority (continuous; in years), number of computer systems used in the department (continuous), and type of education (nominal; bachelor’s or master’s degree or higher).

**Workload** was assessed using a shortened version of the Expanding Nursing Stress Scale (ENSS) by French et al. [38]. The original questionnaire examines various stressors in nurses’ work and consists of 57 items. One factor in the questionnaire deals with workload. It consists of nine items, and its reliability was α = 0.86. Two of the questionnaire items were not relevant to the topic of the current study and were removed. Therefore, the final shortened version includes seven items on a Likert scale ranging from 1 (“disagree at all” / “very low”) to 6 (“agree to a large extent” / “very high”). An example of an item from the questionnaire: “I don’t have enough time to do what I am required to do.” The workload index was created by averaging the score of all seven items. A higher score means a higher workload. The questionnaire was translated into Hebrew and was validated through a pilot study among a small number of subjects (*N* = 32). Reliability as internal consistency (Cronbach’s alpha) in the pilot phase was 0.955 for the before questionnaire and 0.838 for the after questionnaire. In the final sample, the workload index’s Cronbach’s alpha value was 0.91 (before the implementation of EMR) and 0.86 (after).

**Information availability** was assessed using a shortened version of the Carrington-Gephart Unintended Consequences of Electronic Health Record Questionnaire (CG-UCE-Q) by Gephart et al. [39]. The internal reliability of the original questionnaire was α = 0.94 with a content validity index of 0.96. The original questionnaire consists of 36 questions and covers a variety of topics related to the change in the work process due to the implementation of computerized systems in hospitals. One of the issues covered is the availability of the patient’s medical information. For the present study, we selected five items relevant to measuring the availability of information. The rest of the items in the questionnaire were not relevant to the subject of the current study. Items range on a Likert scale from 1 (“do not agree at all”) to 5 (“strongly agree”). Example item: “When you have to make a decision about your patient, is there too little documented information about the patient for you to understand the clinical picture?“. The medical information availability index was calculated as the average of items’ scores after reversing the scores of items #1, 4, and 5. A higher score means greater availability of information. The questionnaire was translated into Hebrew and was validated among a small number of subjects (*N* = 32) from the study population. Reliability as internal consistency (Cronbach’s alpha) in the pilot phase was 0.924 for the before EMR questionnaire and 0.779 for the after questionnaire. In the final sample, the index’s Cronbach’s alpha value was 0.690 before EMR and 0.930 after.

**Medication errors** were assessed using a shortened version of the Medical Error Checklists questionnaire developed by Tsiga et al. [40]. The internal reliability of the original questionnaire was α = 0.96. The original tool is constructed of three parts, with each part containing 25 questions. The items represent a variety of medical errors, for example, wrong diagnosis, errors in medication prescriptions, communication failure, etc. Only eight items were found to be relevant for the current study, measuring medication administration errors, and those comprised the final tool used in this study. Items range on a Likert scale from 1 (“do not agree at all”) to 5 (“strongly agree”). Example item: “The prescription of the medication is illegible and unclear.” The medication errors index was calculated as the average of all eight items. A higher score means more errors in administering medication. The questionnaire was translated into Hebrew and was validated among a small number of subjects (*N* = 32). Reliability as internal consistency (Cronbach’s alpha) in the pilot phase was 0.954 for the before EMR questionnaire and 0.903 for the after questionnaire. In the final sample, the index’s Cronbach’s alpha value was 0.820 before the implementation of EMR systems and 0.700 after.

### Statistical analysis

The statistical analysis of the results was performed using SPSS Version 28. The analysis included both descriptive and analytic statistics to explore the research hypotheses. This study has no missing data handling due to all items on the questionnaire being mandatory to answer. The statistical tests were chosen according to the variable distributions. Given the large sample size, parametric tests were used even for non-normally distributed measurements. Correlation between continuous variables was assessed using the Pearson correlation test. Associations between categorical and continuous variables were examined using Student’s paired-samples t-test. Multivariate regression analysis was conducted using the linear regression model for all three main dependent variables (medication errors, workload, and medical information availability). Analyses were performed in Enter mode following the negation of multi-collinearity. Only variables found to be associated with the dependent variables in the univariate analysis were introduced into the regression analyses. A *p*-value of 0.05 or lower was deemed statistically significant in all statistical analyses.

## Results

### Sample description

In total, 622 nurses entered the questionnaire link, of which 31 (5%) indicated that they did not use pen-and-paper medical records and were subsequently excluded from the rest of the study. The final sample included 591 registered nurses working in government hospitals in Israel, of which 148 men (25%) and 443 women (75%). The average age was 33.92 years (SD 9.24 years), with a median age of 30.5. Most participants were employees at the ‘Sorasky’ Medical Center in Tel Aviv and “Hillel Yaffe” Hospital in Hadera (105 and 327, respectively). See Table 1 for additional socio-demographic breakdown.

**Table 1:** Socio-demographic breakdown of studied sample (*N* = 591)

Variable	N (%) / Mean (± SD)
**Gender**	
Female	443 (75.0%)
Male	148 (25.0%)
**Family status**	
Coupled with children	208 (35.8%)
Coupled without children	159 (27.4%)
Single with children	205 (35.3%)
Single without children	9 (1.5%)
**Role**	
Registered nurse	558 (94.4%)
Head nurse	16 (2.7%)
Deputy head nurse	17 (2.9%)
**Department**	
Intensive care unit	105 (17.2%)
Surgery	175 (28.6%)
Emergency department	116 (19.0%)
Internal medicine	77 (12.6%)
Cardiology	22 (3.6%)
Other	117 (19.0%)
**Age (years)**	33.92 (± 9.24)
**Number of EMR systems used at work**	2.42 (± 1.21)
**Seniority (years)**	7.68 (± 7.62)

EMR = Electronic Medical Record

### Quantitative analysis

The findings show a significant difference in all assessed indices when comparing before the implementation of EMR systems and after, as follows. The perception of the number of medication errors after EMR systems implementation (M = 2.2, SD = 0.72) was reduced compared to before (M = 3.12, SD = 0.73) (t = 24.85, *p* < 0.001). The workload after implementation of EMR systems (M = 2.77, SD = 0.92) was perceived as lower compared to before (M = 3.6, SD = 1.07) (t = 15.53, *p* < 0.001). In total, this represents a ∼ 30% decrease in both medication errors and workload perception following the introduction of EMR systems. However, in contrast to our hypothesis, medical information availability after the implementation of EMR systems (M = 2.45, SD = 1.35) was lower compared to before (M = 2.6, SD = 0.74) (t = 2.44, p. 0.015). In total, this represents a ∼ 10% decrease in information availability following the introduction of EMR systems.

In order to examine the relationship between socio-demographic variables and the main variables, as a first step, a univariate analysis was performed to explore the association with attitudes after the implementation of the EMR. None of the Demographic variables were significant for workload and medication errors (*p* > 0.05), and all of them were significant for information availability (*p* < 0.001) (see Table 2).

**Table 2:** Differences in perception of (a) workload, (b) medication errors, and (c) Medical information availability after the implementation of Electronic Medical Records (EMR) across socio-demographic categories (*N* = 591)

Variable	Categories	M (SD)	*p*-value
**(a) Workload**			
Gender	Women	2.73 (0.88)	0.118
	Men	2.87 (0.96)	
Seniority	Under 5 years	2.71 (0.81)	0.211
	5 years or more	2.81 (0.97)	
Department	Surgical	2.75 (0.88)	0.061
	Internal	2.99 (1.13)	
	Intensive care/emergency department	2.73 (0.84)	
**(b) Medication errors**			
Gender	Women	2.16 (6.6)	0.068
	Men	2.3 (0.87)	
Seniority	Under 5 years	2.18 (0.65)	0.741
	5 years or more	2.2 (0.78)	
Department	Surgical	2.13 (0.68)	0.293
	Internal	2.19 (0.9)	
	Intensive care/emergency department	2.23 (0.69)	
**(c) Medical information availability**			
Gender	Women	2.3 (1.35)	< 0.001
	Men	2.9 (1.27)	
Seniority	Under 5 years	2.08 (1.26)	< 0.001
	5 years or more	2.84 (1.35)	
Department	Surgical	2.5 (1.46)	< 0.001
	Internal	3.6 (0.66)	
	Intensive care/emergency department	2.17 (1.28)	

Note: *p*-value computed using t-test for gender and seniority and one-way ANOVA for department

For each of the primary dependent variables, a delta score was computed by subtracting the value before EMR systems implementation from the value after. The mean for the delta score of medication errors was − 0.92 (SD 0.90), -0.83 (SD 1.03) for workload, and − 0.14 (SD 0.39) for medical information availability. These delta scores were used for correlation analyses. The results here show that the delta score of workload was positively correlated with the delta score of medical information availability (*r* = 0.21, *p* < 0.01) and medication errors (*r* = 0.36, *p* < 0.01). In other words, a higher increase in workload was associated with more errors and higher availability of information, or vice versa. In addition, the delta score of medication errors was negatively associated with the delta score of medical information availability (*r*=-0.37, *p* < 0.01), meaning that fewer medication errors are reported with the increase in medical information availability.

Finally, multivariate linear regression was conducted for each of the three main dependent variables separately - medication errors, workload, and information availability. The analysis was performed to predict the dependent variables after implementation of EMR systems. See the complete results in Table 3.

**Table 3:** Regression analysis of main variables after the implementation of Electronic Medical Records (EMR) (*N* = 591)

Main variables assessed	Medical Information Availability	Medication Errors	Workload
**F (** ***p*** **-value)**	26.16 (< 0.001)	5.77 (< 0.001)	5.30 (< 0.01)
**R²**	15.2	5.7	2.6
**Maximum VIF**	< 1.1	< 1.1	< 1.1
**Residuals distribution**	[2.078–2.710]	[2.347–3.237]	[2.149–4.047]
**Cook’s distance**	[0.000–0.019]	[0.000–0.022]	[0.000–0.055]
**Unstandardized and standardized coefficients– B (β)**			
Age	---	-0.003 (-0.033)	0.003 (0.032)
Gender	-0.587 (-0.188)^***^	-0.146, (0.088)^*^	-0.177 (-0.086)^*^
Attitude before EMR implementation	0.369 (0.202)^***^	0.218, (0.220)^***^	0.124 (0.148)^***^
Department	-0.675 (-0.246)^***^	---	---

Note: Regression performed in Enter mode following negation of multi-collinearity (see maximum VIF values reported in the table). Age and attitude prior to EMR implementation were entered as continuous variables; Gender (0 = female, 1 = male); Department (0 = Emergency or intensive care, 1 = other)

^*^p-value < 0.05 (two-folds) ^***^p-value < 0.001 (two-folds)

### Qualitative analysis

In the qualitative section of the research, 82 participants answered three open questions (see methodology). The purpose of the questions was to understand how the nursing staff experienced using EMR systems and its effect on their work. It is important to note that there were contrasting reports in answers; for some participants, a certain feature was a disadvantage, but for other participants, it was an advantage. For example, while one participant claimed that EMR wastes a lot of time in her work, another claimed that it saves her a lot of time. It is also interesting to note that in response to the question about the EMR systems’ benefits, only a few participants reported a decrease in medication administration errors as a response. In the analysis, we categorized several themes that appeared repeatedly in the answers, as will be specified below.

#### Theme #1: additional workload that comes at the expense of patient care time

A large portion of participants (28 out of 82 who responded to the questions) claimed that the EMR systems require them to devote time to operate them at the expense of time for actual care for patients, such as giving support, communicating with the patient, and responding to their needs, especially when compared to the era of pen and paper prescriptions before EMR implementation. For example - “more information about the patients, less time for nursing care”; “It (EMR system) is excellent but leaves less time to treat the patient physically and emotionally”; “Filling out multiple indices and a lot of screen time that should have been used as a quality time with the patient bothers me.” In fact, some of the participants said that EMR systems add to their workload; for example -“Using information systems has added a lot of additional tasks to our work and at the same time, no personnel has been added; the same number of nurses remain on shifts, which makes me working under stressful conditions because you have to complete both the work in front of the computer and the work with the patient and the family.”

In this context, some participants also complained about the requirement to electronically document many details and indices about the patient that, in their opinion, have no medical meaning. Participants said that many unnecessary indices need to be entered into the system due to various administrative requirements and not for medical necessity.

#### Theme # 2: limitations and technical faults of the systems

Another kind of feedback was related to technical problems or slowness of the systems, for example - “Only the quality of the system and the equipment disturbs me; for example, the slow transition between windows”); Communication failures between systems, connection problems due to internet connection dependency, for example - “very interrupting when there is no Internet access”), and problems during a power outage or the collapse of the system - “Computing failures. System collapse. Power failures. Software failures. Too many versions”; “Everything is fine and lovely until there is a power outage; computer crashes; slow computer. It is just terrible. In the age of paper records, there was less writing and less information. But the basic important information was there. I do not recommend going back to paper, but there’s a need to take care of a good backup system with the necessary information that will come into action in the event of a malfunction”. Participants wrote about other computer malfunctions, such as bugs in the systems that cause them to get stuck and waste a lot of time as a result, for example - “Using Computers is excellent. But the Chameleon system has a lot of bugs and often gets stuck. Wastes a lot of time during the shift…”.

#### Theme # 3: the human factor as a source of problems in the operation of EMR systems

Finally, a theme related to the human factor emerged from participants’ feedback, namely the use of computerized systems by the medical and nursing staff. It was claimed that the physicians do not enter the medical instructions into the system in a straightforward manner or that necessary instructions are missing, or critical information about the patients is missing, for example - “… there are still lots of mistakes in writing instructions”; “sometimes the staff does not enter information properly, and sometimes critical information about the patients is missing.” Other nurses reported that their colleagues copy data from each other to save time filling them out, for example - “because it takes more time to fill out indices, many times poor indices are copied from nurse to nurse instead of filling out the correct data”). Others stated that older staff members have difficulty acquiring skills for using the EMR systems due lower digital literacy, for example - “You need full control in computer skills, which makes it difficult for older nurses to use them (EMR systems)”; “It is important to note that there are older employees who do not get along with the computer and it will be difficult for them to work and use the computer. This has a negative effect on the work of others in the department”).

## Discussion

The current study examined the effect of implementing EMR systems on the extent of errors in medication administration by qualified nursing staff and on other variables related to patient safety, namely the availability of medical information and the workload imposed on nursing staff. In line with the study hypotheses, we found that EMR systems reduce errors in the administration of medications and reduce workload. These findings correspond with the prevailing position in the literature on the effectiveness of EMR systems in reducing errors in prescriptions and medication administration [11, 41]. It is important to emphasize that, unlike most studies on this topic, the extent of errors in the present study was measured based on an approximate perception of nursing staff and not as an exact quantitative measure. However, similar approaches in the literature are reported [42].

The findings suggest that workload decreased for the most part following the implementation of EMR systems. This decrease in workload can be explained by the fact that computerized systems save the need to physically run around to receive and transfer various materials such as laboratory tests, imaging, drug prescriptions, etc [43]... Nevertheless, the findings suggest that there is also a disadvantage. According to some participants’ reports, EMR systems require a lot of handling time, which comes at the expense of care for the patients and their families. This finding partially aligns with the findings of a meta-analysis performed by Moore et al. [44], which concluded that computerized medical systems increase the time nurses spent documenting medical records. Furthermore, in the same meta-analysis, it was found that even after the implementation of the EMR systems, there were nurses who preferred to continue documenting manually and viewing the older pen-and-paper method as faster and more accesible.

However, other studies included in Moore’s meta-analysis [44] claim that EMR systems contributed to the redistribution of nurses’ working time so that they devoted more time to direct patient care and communication with family members than dealing with medical records. These studies further claim that this resulted in greater satisfaction and a sense of meaning in their work. Arguably, the findings of the current study contradict this. It is possible that part of this discrepancy in findings can be explained by the type of system used since the type of EMR system has a decisive effect on the required documentation time [43]. Other studies also found, in line with the current study, that EMR systems harm the workflow of the team since they require multi-tasking, distract nurses from their primary work, and reduce the contact and interpersonal relationship with patients, negatively affecting the satisfaction of both the patients and the staff [45]. Another support for the findings of this current research is found in a study among doctors in the USA who claimed that one of the main reasons for burnout of doctors, which often results in leaving the job, is the need to spend too much time documenting information in EMR systems [46].

There may be a need to find ways to reduce the required documentation during a shift and document only vital medical information. This is important to avoid a situation where nurses devore extended periods of time during their shifts sitting in front of the computer instead of providing care and attention to patients and communicating with families. It should be noted that although the workload in this study is subjective and relies on the nursing staff’s report, it is, in fact, a preferred measurement for this topic [43].

In contrast to our hypothesis, medical information availability dropped after implementing EMR systems. These findings are slightly surprising as most studies on the subject found an improvement in the availability of medical information following EMR systems [32]. This finding may also highlight some of the backlash reported by nurses concerning the difficulty of managing the work with EMR systems during their shifts.

Alongside this, we found that other variables can explain part of the reduction in information availability. For example, nurses responding to our questionnaire who are working in the Intensive care and emergency departments reported significantly less information availability after the introduction of EMR systems. Arguably, in departments dealing with urgent or intense cases, there is a greater need for high-speed information transferring [43]. It may be critical if the EMR system is slow or gets stuck, as is sometimes the case with such systems and as reported by some of the nurses. Indeed, the literature accounts for technical deficiencies of systems, such as slowness, failures, systems crashes, communication problems, and difficulty integrating between the EMR system and others [32]. Moreover, the literature reports that EMR systems sometimes contain too many complicated and less vital functions [32]. It may be necessary to tailor the computerized information systems specifically to these departments so that they offer a “lean” or easier interface that will allow for faster extraction of vital medical information. It is also a good idea to make sure that there is sufficient backing to operate the systems for cases of communication or electricity faults so that crucial information remains available in these situations as well.

The decrease in information availability after EMR implementation can also be explained by insufficient training for staff to use those systems properly. As reported by participants in the qualitative section of the study, departments have elderly staff memebers who have difficulty operating and controlling computerized systems. Similar findings were also found in previous studies [32]. The findings of the present study are in line with the accumulated findings that indicate a fundamental need to think and redesign some of the EMR systems from the perspective of the end users, as well as to provide appropriate training and support for using them.

### Limitations and future directions

This study has several limitations. First, the sampling method in the current study was not probabilistic and was based on convenience and the snowball method exposed the sample to selection bias and may not fully represent the population. Second, the dependent variables were measured subjectively, i.e., as an experience or impression of the study participants. Such tools are naturally exposed to biases since the impressions of the respondents do not necessarily accurately reflect the objective reality in the hospitals. Memory bias should be considered for reports concerning the time before EMR systems implementation. In addition, there may be reporting bias due to the unwillingness to report the actual occurrence of medical errors.

Third, this study measured only three variables. Although these are three main variables in understanding the phenomenon being investigated, it must be assumed that additional variables are required to obtain a broader and more detailed picture of the computerized systems in a hospital. It is useful to specifically and directly examine variables such as the quality and accuracy of medical information [32] or the time of documentation of medical information [47]. It is also possible to distinguish between different types of medication errors and examine each of them individually, for example, errors in identifying the patient, dosage, or how the medication is administered.

## Conclusions

This study provides mixed results regarding the research hypotheses. The findings support the hypotheses stating that Electronic Medical Records (EMRs) are perceived by Israeli nurses to reduce medical errors and workload, compared to the older pen-and-paper approach. However, the findings do not support the third hypothesis since the findings show that EMRs were perceived to decrease information availability compared to the pen-and-paper era.

The findings in this study show the great benefits of using EMR systems in hospitals in Israel, as well as the difficulties and challenges associated with them. To the best of our knowledge, no systematic research has yet been done on this subject in the State of Israel, and therefore, the findings of the current study are highly important for decision-makers.

The findings show that the implementation of EMR systems in Israel contributes to reducing errors in the administration of medications by qualified nursing staff from the point of view and the direct experience of the nursing staff themselves. This means that these systems contribute to saving lives, and therefore, their importance for hospitals is tremendous. In addition, according to nurses, the systems reduce the workload imposed on the staff. However, the findings present difficulties on two levels. The first is the need for multi-tasking, which harms nurses’ work. This difficulty is manifested in the fact that too much time is required to document medical information in computerized systems, which comes at the expense of time directly caring for patients and their families. This situation may harm the nurses’ morale and may cause burnout at work. The second level is the complexity and slowness of the systems, which may reduce the availability of medical information when it is necessary to retrieve quickly, which is especially problematic in departments of surgery, intensive care, and emergency medicine, where a lot of medical information is needed urgently and immediately.

The findings raise a need for rethinking and redesigning these systems while thinking about the end users, as well as a need for dedicated training for their use by end-users of different digital literacy backgrounds. Future research can focus on developing methods for assimilating knowledge and skills to use computerized systems in a way that considers age and digital literacy and evaluates their effectiveness. In addition, future research will be able to examine the effectiveness of changes and improvements in the computerized systems, particularly the development of “lean” versions of the interfaces for departments where quick information retrieval is required.

### Electronic supplementary material

Below is the link to the electronic supplementary material.

Supplementary Material 1

## Data Availability

The datasets used and/or analyzed during the current study are available from the corresponding author upon reasonable request.

## Declarations

### Authors’ information

Moran Bodas is a Senior Lecturer at the Department of Emergency & Disaster Management, School of Public Health, Faculty of Medicine, Tel-Aviv University. He is the former director of the National Center for Trauma & Emergency Medicine Research at the Gertner Institute. Raneen Naamneh is a registered nurse who successfully graduated from the School of Public Health after completing her thesis reported in this paper.

## Abbreviations

CG: UCE-Q-Carrington-Gephart Unintended Consequences of Electronic Health Record Questionnaire
EHR: Electronic Health Record
EMR: Electronic Medical Record
ENSS: Expanding Nursing Stress Scale
e: PS-Electronic (medication) Prescription Systems
HMO: Health Management Organization
WHO: World Health Organization

## References

[1] Carver N, Gupta V, Hipskind JE (2023). Medical errors.

[2] Rodziewicz TL, Houseman B, Hipskind JE. Medical error reduction and prevention. 2018. 29763131

[3] Tariq RA, Vashisht R, Sinha A, Scherbak Y. Medication dispensing errors and prevention. 2018. 30085607

[4] Ministry H, Medication management. 2016 update 2016 [ https://www.health.gov.il/hozer/ND117_2016.pdf.

[5] Mieiro DB, Oliveira ÉBCd F, REPd, Mininel VA, Zem-Mascarenhas SH, Machado RC (2019). Strategies to minimize medication errors in emergency units: an integrative review. Revista brasileira de enfermagem.

[6] Scott IA, Pillans PI, Barras M, Morris C (2018). Using EMR-enabled computerized decision support systems to reduce prescribing of potentially inappropriate medications: a narrative review. Therapeutic Adv drug Saf.

[7] Uslu A, Stausberg J (2021). Value of the electronic medical record for hospital care: update from the literature. J Med Internet Res.

[8] Honavar SG. Electronic medical records–The good, the bad and the ugly. Medknow; 2020. pp. 417-8. 10.4103/ijo.IJO_278_20 PMC7043175 32056991

[9] Gopidasan B, Amanullah S, Adebowale A (2022). Electronic medical records–A review of cost-effectiveness, efficiency, quality of care, and usability. J Psychiatry Spectr.

[10] Kadmon G, Bron-Harlev E, Nahum E, Schiller O, Haski G, Shonfeld T (2009). Computerized order entry with limited decision support to prevent prescription errors in a PICU. Pediatrics.

[11] Ammenwerth E, Schnell-Inderst P, Machan C, Siebert U (2008). The effect of electronic prescribing on medication errors and adverse drug events: a systematic review. J Am Med Inform Assoc.

[12] Gates PJ, Hardie R-A, Raban MZ, Li L, Westbrook JI (2021). How effective are electronic medication systems in reducing medication error rates and associated harm among hospital inpatients? A systematic review and meta-analysis. J Am Med Inform Assoc.

[13] Westbrook JI, Baysari MT, Li L, Burke R, Richardson KL, Day RO (2013). The safety of electronic prescribing: manifestations, mechanisms, and rates of system-related errors associated with two commercial systems in hospitals. J Am Med Inform Assoc.

[14] Al-Sarawi F, Polasek TM, Caughey GE, Shakib S (2019). Prescribing errors and adverse drug reaction documentation before and after implementation of e‐prescribing using the Enterprise Patient Administration System. J Pharm Pract Res.

[15] Mills PR, Weidmann AE, Stewart D (2017). Hospital electronic prescribing system implementation impact on discharge information communication and prescribing errors: a before and after study. Eur J Clin Pharmacol.

[16] Aziz MT, Ur-Rehman T, Qureshi S, Bukhari NI (2015). Reduction in chemotherapy order errors with computerised physician order entry and clinical decision support systems. Health Inform Manage J.

[17] Vaidotas M, Yokota PKO, Negrini NMM, Leiderman DBD, Souza VPd S, OFPd et al. Medication errors in emergency departments: is electronic medical record an effective barrier? Einstein (São Paulo). 2019;17. 10.31744/einstein_journal/2019GS4282 PMC6611086 31291385

[18] Hinojosa-Amaya JM, Rodríguez‐García FG, Yeverino‐Castro SG, Sánchez‐Cárdenas M, Villarreal‐Alarcón MÁ, Galarza‐Delgado DÁ (2016). Medication errors: electronic vs. paper‐based prescribing. Experience at a tertiary care university hospital. J Eval Clin Pract.

[19] Hitti E, Tamim H, Bakhti R, Zebian D, Mufarrij A (2017). Impact of internally developed electronic prescription on prescribing errors at discharge from the emergency department. Western J Emerg Med.

[20] Han JE, Rabinovich M, Abraham P, Satyanarayana P, Liao TV, Udoji TN (2016). Effect of electronic health record implementation in critical care on survival and medication errors. Am J Med Sci.

[21] Pettit NN, Han Z, Choksi A, Voas-Marszowski D, Pisano J (2019). Reducing medication errors involving antiretroviral therapy with targeted electronic medical record modifications. AIDS Care.

[22] Hernandez F, Majoul E, Montes-Palacios C, Antignac M, Cherrier B, Doursounian L (2015). An observational study of the impact of a computerized physician order entry system on the rate of medication errors in an orthopaedic surgery unit. PLoS ONE.

[23] Vicente Oliveros N, Gramage Caro T, Pérez Menendez-Conde C, Álvarez‐Díaz AM, Martín‐Aragón Álvarez S, Bermejo Vicedo T (2017). Effect of an electronic medication administration record application on patient safety. J Eval Clin Pract.

[24] Tubaishat A (2019). The effect of electronic health records on patient safety: a qualitative exploratory study. Inform Health Soc Care.

[25] Koppel R, Metlay JP, Cohen A, Abaluck B, Localio AR, Kimmel SE (2005). Role of computerized physician order entry systems in facilitating medication errors. JAMA.

[26] Bell SK, Delbanco T, Elmore JG, Fitzgerald PS, Fossa A, Harcourt K (2020). Frequency and types of patient-reported errors in electronic health record ambulatory care notes. JAMA Netw open.

[27] Classen DC, Holmgren AJ, Newmark LP, Seger D, Danforth M, Bates DW (2020). National trends in the safety performance of electronic health record systems from 2009 to 2018. JAMA Netw open.

[28] Moreland PJ, Gallagher S, Bena JF, Morrison S, Albert NM (2012). Nursing satisfaction with implementation of electronic medication administration record. CIN: Computers Inf Nurs.

[29] Cho KW, Kim SM, An C-H, Chae YM (2015). Diffusion of electronic medical record based public hospital information systems. Healthc Inf Res.

[30] Carvalho DPd, Rocha LP, Pinho ECd, Tomaschewski-Barlem JG, Barlem ELD, Goulart LS (2019). Workloads and burnout of nursing workers. Revista brasileira de enfermagem.

[31] Vaismoradi M, Tella S, Logan A, Khakurel P, Vizcaya-Moreno J (2020). Nurses’ adherence to patient safety principles: a systematic review. Int J Environ Res Public Health.

[32] Tsai CH, Eghdam A, Davoody N, Wright G, Flowerday S, Koch S (2020). Effects of electronic health record implementation and barriers to adoption and use: a scoping review and qualitative analysis of the content. Life.

[33] Dudding KM, Gephart SM, Carrington JM (2018). Neonatal nurses experience unintended consequences and risks to patient safety with electronic health records. CIN: Computers Inf Nurs.

[34] Ministry H. Workplan - Nursing Administration– 2020. 2020.

[35] Culotta A, editor. Reducing sampling bias in social media data for county health inference. Joint Statistical Meetings Proceedings; 2014: Citeseer.

[36] Abramson JH (2011). WINPEPI updated: computer programs for epidemiologists, and their teaching potential. Epidemiol Perspect Innovations.

[37] Shkedi A. Words that try to touch: Qualitative research—Theory and application. Tel-Aviv: Ramot. 2003.

[38] French SE, Lenton R, Walters V, Eyles J (2000). An empirical evaluation of an expanded nursing stress scale. J Nurs Meas.

[39] Gephart SM, Bristol AA, Dye JL, Finley BA, Carrington JM (2016). Validity and reliability of a new measure of nursing experience with unintended consequences of electronic health records. CIN: Computers Inf Nurs.

[40] Tsiga E, Panagopoulou E, Montgomery A (2017). Examining the link between burnout and medical error: a checklist approach. Burnout Res.

[41] Manias E, Kusljic S, Wu A (2020). Interventions to reduce medication errors in adult medical and surgical settings: a systematic review. Ther Adv Drug Saf.

[42] Jindal SK, Raziuddin F (2018). Electronic medical record use and perceived medical error reduction. Int J Qual Service Sci.

[43] Bosman RJ (2009). Impact of computerized information systems on workload in operating room and intensive care unit. Best Pract Res Clin Anaesthesiol.

[44] Moore EC, Tolley CL, Bates DW, Slight SP (2020). A systematic review of the impact of health information technology on nurses’ time. J Am Med Inform Assoc.

[45] Schenk E, Schleyer R, Jones CR, Fincham S, Daratha KB, Monsen KA (2018). Impact of adoption of a Comprehensive Electronic Health record on nursing work and Caring Efficacy. Comput Inf Nurs.

[46] Downing NL, Bates DW, Longhurst CA (2019). Physician burnout in the electronic health record era. Ann Intern Med.

[47] Baumann LA, Baker J, Elshaug AG (2018). The impact of electronic health record systems on clinical documentation times: a systematic review. Health Policy.

