# Supplementary material for: The effect of electronic medical records on medication errors, workload, and medical information availability among qualified nurses in Israel– a cross sectional study
Source: BMC Nurs. 2024 Apr 24;23:270. doi: 10.1186/s12912-024-01936-7 (PMC11044371; doi:10.1186/s12912-024-01936-7)
Supplement: Supplementary file 1 — Supplementary Material 1 [file 12912_2024_1936_MOESM1_ESM.docx]

STROBE Statement—checklist of items that should be included in reports of observational studies

|  | Item No. | Recommendation | Page  No. | Relevant text from manuscript |
| --- | --- | --- | --- | --- |
| **Title and abstract** | 1 | (*a*) Indicate the study’s design with a commonly used term in the title or the abstract | 1 | "A Cross Sectional Study" |
|  |  | (*b*) Provide in the abstract an informative and balanced summary of what was done and what was found | 2 | See abstract |
| Introduction | | | | |
| Background/rationale | 2 | Explain the scientific background and rationale for the investigation being reported | 4-6 |  |
| Objectives | 3 | State specific objectives, including any prespecified hypotheses | 6 | "This current study aimed to assess nurses' perception of EMR systems' contribution to mitigating medical error, workload, and information availability. The working hypotheses were that nurses perceive the introduction of EMR systems as beneficial to reducing medical errors and workload and increasing information availability, compared with the previous pen & paper system." |
| Methods | | | | |
| Study design | 4 | Present key elements of study design early in the paper | 7 | "This cross-sectional study…" |
| Setting | 5 | Describe the setting, locations, and relevant dates, including periods of recruitment, exposure, follow-up, and data collection | 7 | "performed between February and May 2022…" |
| Participants | 6 | (*a*) *Cohort study*—Give the eligibility criteria, and the sources and methods of selection of participants. Describe methods of follow-up  *Case-control study*—Give the eligibility criteria, and the sources and methods of case ascertainment and control selection. Give the rationale for the choice of cases and controls  *Cross-sectional study*—Give the eligibility criteria, and the sources and methods of selection of participants | 7-8 | "The inclusion criteria for this study were being a registered nurse, an adult (over age 18), Hebrew speaking, and having a recollection of the period in which pen-and-paper records were used." |
|  |  | (*b*) *Cohort study*—For matched studies, give matching criteria and number of exposed and unexposed  *Case-control study*—For matched studies, give matching criteria and the number of controls per case | N.A. |  |
| Variables | 7 | Clearly define all outcomes, exposures, predictors, potential confounders, and effect modifiers. Give diagnostic criteria, if applicable | 9-11 | Variables and tools are reported |
| Data sources/ measurement | 8* | For each variable of interest, give sources of data and details of methods of assessment (measurement). Describe comparability of assessment methods if there is more than one group | *9-11* | *e.g., "*… *the Medical Error Checklists questionnaire developed by Tsiga et al. (40). The internal reliability of the original questionnaire was α=0.96."* |
| Bias | 9 | Describe any efforts to address potential sources of bias | 21 | See Limitations clause |
| Study size | 10 | Explain how the study size was arrived at | 8 | " The minimum sample size was calculated using the WinPepi calculator… " |
| Quantitative variables | 11 | Explain how quantitative variables were handled in the analyses. If applicable, describe which groupings were chosen and why | 11-12 | See statistical analysis |
| Statistical methods | 12 | (*a*) Describe all statistical methods, including those used to control for confounding | 9 | See socio-demographics |
|  |  | (*b*) Describe any methods used to examine subgroups and interactions | 11-12 | See statistical analysis |
|  |  | (*c*) Explain how missing data were addressed | N.A. |  |
|  |  | (*d*) *Cohort study*—If applicable, explain how loss to follow-up was addressed  *Case-control study*—If applicable, explain how matching of cases and controls was addressed  *Cross-sectional study*—If applicable, describe analytical methods taking account of sampling strategy | N.A. |  |
|  |  | (*e*) Describe any sensitivity analyses | N.A. |  |
| Results | | | | |
| Participants | 13* | (a) Report numbers of individuals at each stage of study—eg numbers potentially eligible, examined for eligibility, confirmed eligible, included in the study, completing follow-up, and analysed | 13 | See Sample description |
|  |  | (b) Give reasons for non-participation at each stage | 13 | "31 (5%) indicated that they did not use pen-and-paper medical records and were subsequently excluded from the rest of the study." |
|  |  | (c) Consider use of a flow diagram | N.A. |  |
| Descriptive data | 14* | (a) Give characteristics of study participants (eg demographic, clinical, social) and information on exposures and potential confounders | 13 | See Sample description |
|  |  | (b) Indicate number of participants with missing data for each variable of interest | N.A. |  |
|  |  | (c) *Cohort study*—Summarise follow-up time (eg, average and total amount) |  |  |
| Outcome data | 15* | *Cohort study*—Report numbers of outcome events or summary measures over time |  |  |
|  |  | *Case-control study—*Report numbers in each exposure category, or summary measures of exposure |  |  |
|  |  | *Cross-sectional study—*Report numbers of outcome events or summary measures | 13-14 | See Results |
| Main results | 16 | (*a*) Give unadjusted estimates and, if applicable, confounder-adjusted estimates and their precision (eg, 95% confidence interval). Make clear which confounders were adjusted for and why they were included | 13-14 | See Results |
|  |  | (*b*) Report category boundaries when continuous variables were categorized | N.A. |  |
|  |  | (*c*) If relevant, consider translating estimates of relative risk into absolute risk for a meaningful time period | N.A. |  |
| Other analyses | 17 | Report other analyses done—eg analyses of subgroups and interactions, and sensitivity analyses | 15-17 | See "Qualitative analysis" |
| Discussion | | | | |
| Key results | 18 | Summarise key results with reference to study objectives | 18-22 | "In line with the study hypotheses, we found that EMR systems reduce errors in the administration of medications and reduce workload." |
| Limitations | 19 | Discuss limitations of the study, taking into account sources of potential bias or imprecision. Discuss both direction and magnitude of any potential bias | 21 | See Limitations clause |
| Interpretation | 20 | Give a cautious overall interpretation of results considering objectives, limitations, multiplicity of analyses, results from similar studies, and other relevant evidence | 22 | See Conclusions |
| Generalisability | 21 | Discuss the generalisability (external validity) of the study results | 21 | See Limitations clause |
| Other information |  |  | | |
| Funding | 22 | Give the source of funding and the role of the funders for the present study and, if applicable, for the original study on which the present article is based | N.A. |  |

*Give information separately for cases and controls in case-control studies and, if applicable, for exposed and unexposed groups in cohort and cross-sectional studies.

**Note:** An Explanation and Elaboration article discusses each checklist item and gives methodological background and published examples of transparent reporting. The STROBE checklist is best used in conjunction with this article (freely available on the Web sites of PLoS Medicine at http://www.plosmedicine.org/, Annals of Internal Medicine at http://www.annals.org/, and Epidemiology at http://www.epidem.com/). Information on the STROBE Initiative is available at www.strobe-statement.org.
